# Tailoring discharge planning after pediatric febrile urinary tract infection: a cohort study

**DOI:** 10.1007/s00431-026-07479-0

**Published:** 2026-10-13

**Authors:** Pierluigi Marzuillo, Stefano Guarino, Luigi Annicchiarico Petruzzelli, Milena Brugnara, Giusy Capasso, Ciro Corrado, Anna Di Sessa, Gabriele Malgieri, Marco Pennesi, Giulio Rivetti, Floriana Scozzola, Francesca Taroni, Paola Tirelli, Claudio La Scola, Andrea Pasini, Giovanni Montini

**Affiliations:** 1 Department of Woman, Child and of General and Specialized Surgery, Università Degli Studi Della Campania “Luigi Vanvitelli”, Via Luigi De Crecchio 2, 80138 Naples, Italy; 2 Pediatric Nephrology and Dialysis Unit, Santobono Children’s Hospital, Naples, Italy; 3 Pediatria C, Ospedale Donna Bambino, Verona, Italy; 4 Pediatric Nephrology, “G. Di Cristina” Hospital, Palermo, Italy; 5 Institute for Maternal and Child Health-IRCCS Burlo Garofolo, Trieste, Italy; 6 Pediatric Unit, Ca’ Foncello Civil Hospital, Treviso, Italy; 7 Pediatric Nephrology, Dialysis and Transplant Unit. Fondazione Ca’ Granda IRCCS, Policlinico Di Milano, Milan, Italy; 8 Pediatric Nephrology and Dialysis Unit, IRCCS Azienda Ospedaliero-Universitaria Di Bologna, Bologna, Italy; 9 Giuliana and Bernardo Caprotti Chair of Pediatrics, Department of Clinical Sciences and Community Health, University of Milan, Milan, Italy

**Keywords:** Congenital anomalies of the kidney and urinary tract, Acute kidney injury, Children, Febrile urinary tract infection

## Abstract

Febrile urinary tract infections (fUTIs) are a common cause of hospitalization in children and may recur in predisposed patients. Although current recommendations define indications for imaging and specialist referral, limited guidance is available to help hospital pediatricians identify, during hospitalization, children with clinical features associated with recurrent fUTI and tailor discharge planning accordingly. We conducted a retrospective, nationwide Italian cohort study including children aged 2 months to 3 years who were hospitalized for fUTI. Routinely collected clinical, laboratory, microbiological and imaging data were analyzed. Patients were stratified by sex and by the presence or absence of previously diagnosed congenital anomalies of the kidney and urinary tract (CAKUT). Adjusted multivariable logistic regression analyses were performed to identify factors associated with recurrent fUTIs. A total of 570 children were included, with a median age of 0.55 years. In the overall cohort, recurrent fUTIs were independently associated with older age, previously diagnosed CAKUT, acute kidney injury (AKI), and hydronephrosis with anterior–posterior diameter of the pelvis 5–15 mm. Among males with previously diagnosed CAKUT, recurrent fUTIs were independently associated with phimosis. Among males without previously diagnosed CAKUT, they were independently associated with AKI and non-*E. coli* infection. Among females, AKI was independently associated with recurrent fUTIs regardless of CAKUT status.

*Conclusions*: In children hospitalized for fUTIs, readily available clinical, laboratory, microbiological, and imaging findings are associated with recurrent fUTIs. Considering these elements during hospitalization may help hospital pediatricians identify children who may benefit from targeted interventions and tailored discharge planning.

**Table:** 

**What is Known:** • *Current Italian recommendations provide guidance on imaging and specialist referral after pediatric febrile urinary tract infection (fUTI) but offer limited guidance for discharge planning.* • *Several clinical factors are associated with recurrent fUTI.*
**What is New:** • *In boys, recurrent fUTIs were independently associated with phimosis in those with known CAKUT and with AKI and non-E. coli infection in those without known CAKUT, whereas in girls AKI was the only independent factor regardless of CAKUT status.* • *These findings were translated into a practical framework to support tailored discharge planning for hospital pediatricians.*

**Supplementary Information:**

The online version contains supplementary material available at https://doi.org/10.1007/s00431-026-07479-0.

## Introduction

Febrile urinary tract infections (fUTIs) are among the most common serious bacterial infections requiring hospitalization in children, accounting for approximately 1.8% of all pediatric admissions [1–3]. Several factors may predispose children to fUTIs and up to one-third of affected patients have an underlying congenital anomaly of the kidney and urinary tract (CAKUT) [1, 4], which significantly increases the risk of recurrence and long-term kidney damage [2].

In routine inpatient practice, children hospitalized with fUTIs are primarily managed by hospital pediatricians, who are responsible not only for acute treatment but also for discharge planning. Existing recommendations identify several established features associated with recurrent fUTIs, particularly CAKUT, abnormal ultrasound findings, and infections caused by organisms other than *E. coli*, and provide guidance regarding imaging and nephro-urological referral [5–7]. However, they are not specifically intended to provide a comprehensive, discharge-oriented framework for hospital pediatricians. Consequently, the practical integration of findings obtained during hospitalization into decisions concerning interim management, prophylaxis, and modifiable clinical factors remains incompletely defined.

Addressing this gap is highly relevant to inpatient pediatric practice. Therefore, adopting the perspective of a hospital pediatrician, we investigated whether additional factors readily identifiable during hospitalization but not specifically emphasized in existing recommendations [5–7], were associated with a history of recurrent fUTIs and could therefore provide clinically relevant information for discharge planning.

## Methods

As previously reported, in a retrospective nationwide Italian cohort study of 849 children, we collected data on all children discharged with a primary diagnosis of fUTI between January 1 st, 2017, and December 31st, 2021 [8]. Children were eligible if they were between birth and 18 years of age and had available data on serum creatinine levels (measured at admission), serum electrolytes, complete blood count, inflammatory markers, kidney ultrasound findings, and previous voiding cystourethrography results when performed according to available recommendations. Patients were managed according to the Italian fUTI recommendations [6, 9].

Kidney ultrasound data refer to examinations performed during hospitalization or according to the timing indicated in the recommendations (2–4 weeks after fUTI) [6, 9]. To align with the age range specified in these recommendations [6, 9] and to address the lack of discharge guidance for this group, we limited the present study to children aged 2 months to 3 years (*n* = 570).

### Definitions

In the study cohort, fUTI was defined as the presence of urinary leukocytes, with or without nitrites, a positive urine culture for a single pathogen, and a body temperature greater than 38 °C in the absence of other clinical signs or symptoms [8].

Patients were classified as having recurrent fUTIs if they had experienced at least two separate fUTI episodes, including the episode that led to hospitalization. Acute kidney injury (AKI) was defined as previously described [8]. The highest-to-baseline serum creatinine ratio was recorded as a continuous measure of kidney function changes during the fUTI episode and contributed to AKI ascertainment.

Chronic kidney disease was defined as either persistent albuminuria (albumin-to-creatinine ratio ≥ 30 mg/g for > 3 months, confirmed in three separate samples) or reduced kidney function, defined as an estimated glomerular filtration rate (eGFR) < 90 mL/min/1.73 m^2^ for > 3 months in children aged > 2 years or below the age-specific reference range in those aged < 2 years [10, 11]. We used the Hoste (age) equation to calculate eGFR [12].

The ultrasound anomalies recorded and presented in Table 1 have been defined elsewhere [6, 8, 9].

**Table 1:** General characteristics of the patients with and without recurrent fUTIs

	Recurrent fUTIs (Yes) No. = 127	Recurrent fUTIs (No) No. = 443	*p*
Age, years, mean (SDS)	1.3 (1.0)	0.83 (0.82)	** < **0.001
Female, No. (%)	59 (46.5)	206 (46.5)	0.99
Birth weight, kg, median (IQR)	3.2 (0.65)	3.2 (0.66)	0.71
Preterm birth, No. (%)	22 (17.3)	69 (15.6)	0.64
Previously known CAKUT, No. (%)	47 (37)	35 (7.9)	** < **0.001
CKD, No. (%)	6 (4.7)	6 (1.4)	0.02
Antibiotic prophylaxis No. (%)	44 (34.6)	0 (0)	< 0.001
Phimosis*, No. (%)	23 (33.8)	42 (17.7)	0.004
Highest/basal serum creatinine ratio, median (IQR)	1.1 (0.65)	0.86 (0.42)	** < **0.001
AKI, No. (%)	37 (29.1)	33 (7.4)	** < **0.001
Na, mEq/L, mean (SDS)	136.3 (3.2)	136.3 (3.0)	0.99
Hyponatremia, No. (%)	34 (26.8)	101 (22.8)	0.35
Hypernatremia, No.(%)	0 (0)	2 (0.5)	0.99
K, mEq/L, mean (SDS)	4.6 (0.63)	4.8 (0.62)	0.002
Hyperkalemia, No. (%)	5 (3.9)	28 (6.3)	0.31
Hypokalemia, No. (%)	0 (0)	3 (0.7)	0.99
Hyponatremia and hyperkalemia, No. (%)	4 (3.1)	11 (2.5)	0.75
WBC, n/mcL, median (IQR)	15,630 (11,102)	16,160 (8510)	0.69
Neutrophils n/mcL, median (IQR)	8745 (7665)	8540 (6690)	0.32
CRP, mg/dL, mean (SDS)	9.7 (9.4)	8.6 (8.1)	0.20
Platelets, n/mcL, median (IQR)	370,000 (187,500)	408,000 (200,000)	0.01
Hydronephrosis (APD 5–15 mm), No. (%)	23 (18.1)	43 (9.7)	0.009
Hydronephrosis (APD > 15 mm), No. (%)	20 (15.7)	35 (7.9)	0.008
Megaureter, No. (%)	31 (24.4)	42 (9.5)	** < **0.001
Unilateral or bilateral kidney Hypodysplasia, No. (%)	18 (14.2)	29 (6.5)	0.006
Bladder abnormalities, No. (%)	2 (1.6)	3 (0.7)	0.31
Non-*E. coli* infection, No. (%)	43 (33.9)	70 (15.8)	** <** 0.001

*AKI* Acute kidney injury, *APD* Anterior–posterior diameter, *CAKUT* Congenital anomalies of the kidney and urinary tract, *CKD* Chronic kidney disease, *CRP* C-reactive protein, *fUTIs* Febrile urinary tract infections, *IQR* Interquartile range, *Na* Sodium,* K* Potassium, *SDS* Standard deviation score, *WBC* White blood cells

*Calculated only considering male patients

Previously known CAKUT was defined as a congenital anomaly of the kidneys or urinary tract documented before the hospitalization considered in this study.

### Statistical analysis

Continuous variables were analyzed using the independent-samples *t*-test for normally distributed data and the Mann–Whitney test for non-normally distributed data. Categorical variables were compared using the chi-square test or Fisher’s exact test, as appropriate.

#### Logistic regression analysis of the overall cohort

Variables significantly associated with recurrent fUTIs in the comparisons reported in Table 1 were first examined in univariable logistic regression analyses. Age and sex were included a priori in the multivariable model because of their clinical relevance and potential confounding effects.

The remaining variables were selected based on their clinical relevance and their association with recurrent fUTIs in the univariable analyses. Antibiotic prophylaxis before hospitalization was excluded because it was reported exclusively among patients with recurrent fUTIs, resulting in complete separation. Phimosis was not included because it is applicable only to males and was evaluated separately in the sex-stratified analyses. The highest-to-baseline serum creatinine ratio was excluded because of its collinearity with AKI. Serum potassium levels and platelet count were also excluded because the observed differences were not considered clinically meaningful or relevant to discharge-oriented risk assessment. Furthermore, the prevalence of hyperkalemia and hypokalemia did not differ between patients with and without recurrent fUTIs. AKI was examined because it is readily identifiable during hospitalization for fUTI and may reflect reduced renal functional reserve, reduced nephron mass, greater kidney involvement during infection, or an underlying urinary tract abnormality [2].

#### Logistic regression analyses stratified by sex and previously known CAKUT status

Additional age-adjusted multivariable logistic regression analyses were performed within each of the four subgroups defined by sex and previously known CAKUT status.

Candidate variables were included if they were significantly associated with recurrent fUTIs in the corresponding subgroup analyses reported in Supplementary Tables 1 and 2.

The general exclusion criteria described above were also applied to these analyses. In males with previously known CAKUT, serum potassium levels were excluded because the mean values were within the normal range, the prevalence of hyperkalemia and hypokalemia did not differ between patients with and without recurrent fUTIs, and the observed difference in mean potassium levels was not considered clinically meaningful or relevant to discharge-oriented risk assessment. In females without previously known CAKUT, the highest-to-baseline serum creatinine ratio was excluded because it contributed to AKI ascertainment and showed high collinearity with the AKI variable.

All statistical analyses were performed using SPSS version 25 for Mac (IBM Corp., Armonk, NY, USA).

## Results

### Overall cohort

Of the original cohort of 849 children, 279 were excluded because they were older than 3 years. Therefore, a total of 570 patients were included, with a median age of 0.55 years (interquartile range 0.29–1.2). Patients with recurrent fUTIs were significantly older and showed a higher prevalence of previously known CAKUT, chronic kidney disease, antibiotic prophylaxis, phimosis among males, AKI, hydronephrosis, megaureter, unilateral or bilateral kidney hypodysplasia, and non-*E. coli* infections compared with patients with no recurrent fUTIs. They also had higher highest-to-baseline serum creatinine ratios, as well as lower serum potassium levels and platelet counts (Table 1).

Among the 570 enrolled patients, 69 developed AKI. Fifty-three had KDIGO stage 1 AKI, 13 had stage 2 AKI, and 3 had stage 3 AKI.

**Fig. 1:**
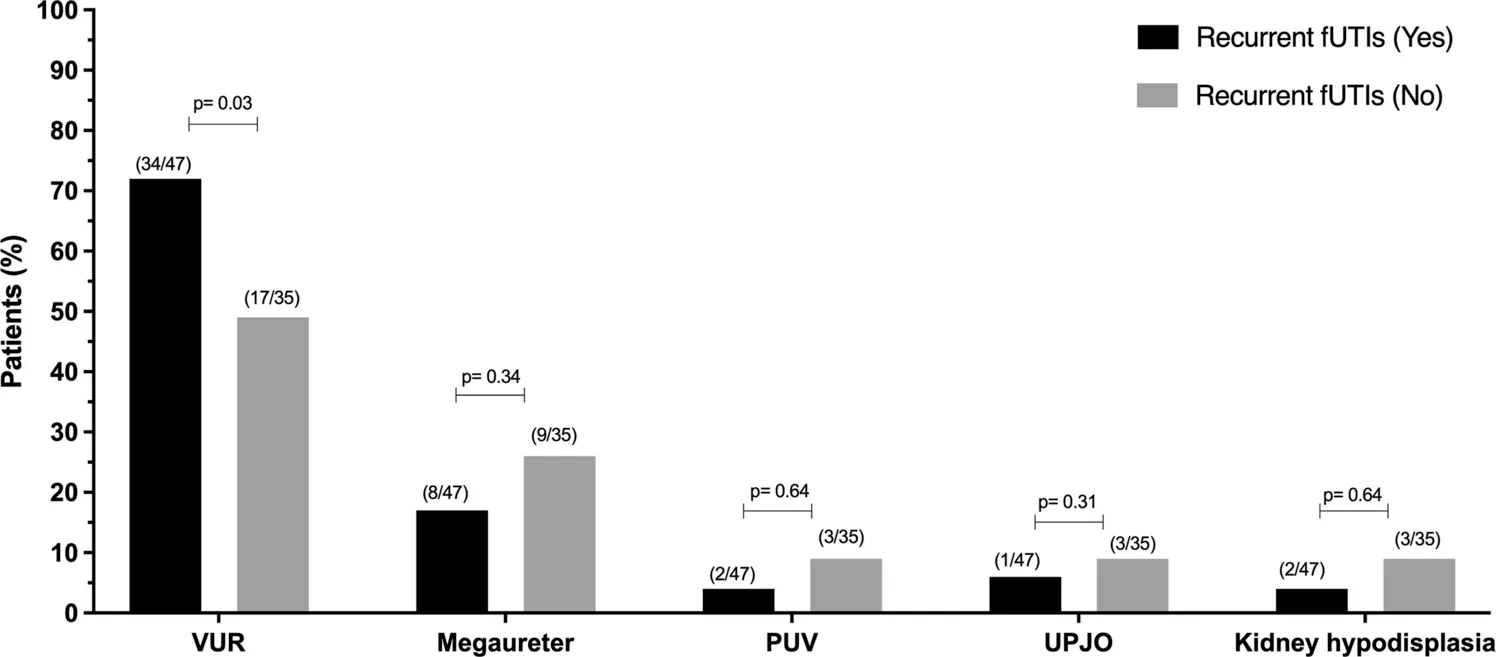
Distribution of individual CAKUT diagnoses among patients with and without recurrent fUTIs. Abbreviations: PUV, posterior urethral valves; UPJO, ureteropelvic junction obstruction; VUR, vesico-ureteral reflux

Figure 1 shows the distribution of previously known CAKUT among patients with and without recurrent fUTIs. Previously known VUR was more prevalent among patients with recurrent fUTIs than among those without recurrence.

In univariate logistic regression analysis of the overall cohort, older age, already known CAKUT, CKD, AKI, hydronephrosis with an APD of 5–15 or > 15 mm, megaureter, unilateral or bilateral kidney hypodysplasia, and non-*E. coli* infections were all associated with recurrent fUTIs (Table 2). In the multivariable logistic regression analysis, only older age (OR 1.45 per 1-year increase, 95% CI 1.14–1.84; *p* = 0.002), previously known CAKUT (OR 4.94, 95% CI 2.64–9.22; *p* < 0.001), AKI (OR 3.09, 95% CI 1.66–5.76; *p* < 0.001), and hydronephrosis with an APD of 5–15 mm (OR 2.00, 95% CI 1.04–3.87; *﻿p* = 0.04) remained independently associated with recurrent fUTIs (Table 2).

**Table 2:** Univariate and multivariable logistic regression analysis of factors associated with recurrent fUTI in the overall cohort

	Univariate			Multiple		
	OR	95% CI	*p*	OR	95% CI	*p*
Age, per 1 year increase	1.6	1.3–2.1	< 0.001	1.5	1.1–1.8	0.002
Male	1.0	0.7–1.5	0.99	0.9	0.58–1.50	0.71
Previously known CAKUT	6.8	4.2–11.3	< 0.001	4.9	2.6–9.2	< 0.001
CKD	3.6	1.1–11.4	0.03	2.3	0.6–9.7	0.24
AKI	5.1	3.0–8.6	< 0.001	3.0	1.7–5.7	< 0.001
Hydronephrosis (APD 5–15 mm)	2.1	1.2–3.6	0.01	2.0	1.1–3.9	0.04
Hydronephrosis (APD > 15 mm)	2.2	1.2–3.9	0.01	1.3	0.6–2.8	0.52
Megaureter	3.1	1.8–5.2	< 0.001	1.1	0.5–2.1	0.88
Unilateral or bilateral kidney hypodysplasia	2.4	1.3–4.4	0.007	0.8	0.3–1.7	0.49
Bladder abnormalities	2.4	0.4–14.2	0.35	–	–	–
Non-*E. coli* infection	2.7	1.7–4.3	< 0.001	1.3	0.8–2.3	0.33

*AKI* Acute kidney injury, *APD* Anterior–posterior diameter, *CAKUT* Congenital anomalies of the kidney and urinary tract, *CI* Confidence interval, *CKD* Chronic kidney disease, *fUTI* Febrile urinary tract infection, *OR* Odds ratio

### Analyses stratified by sex

When the analysis was limited to male patients (Supplementary Table 1), among those with previously known CAKUT recurrent fUTIs were associated with a higher prevalence of antibiotic prophylaxis before admission and phimosis. These patients also had lower serum potassium levels than those without recurrent fUTIs. In contrast, among males without previously known CAKUT, patients with recurrent fUTIs were older and more frequently receiving antibiotic prophylaxis before admission, and had a higher prevalence of AKI, megaureter, and non-*E. coli* infections.

Among female patients (Supplementary Table 2), recurrent fUTIs in those with previously known CAKUT were associated with a higher prevalence of AKI. Among females without previously known CAKUT, those with recurrent fUTIs had a higher prevalence of antibiotic prophylaxis before admission and AKI and showed higher highest-to-baseline serum creatinine ratios compared with patients without recurrent fUTIs.

In the multiple logistic regression analysis (Table 3), recurrent fUTIs were independently associated, among males, with phimosis in those with previously known CAKUT, and with AKI and non-*E. coli* infection in those without previously known CAKUT. Among females, AKI was independently associated with recurrent fUTIs regardless of CAKUT status.

**Table 3:** Multiple logistic regression of factors associated with recurrent fUTIs, stratified by sex and adjusted for age

	Male patients with previously known CAKUT		
	OR	95%CI	*p*
Phimosis	5.1	1.2–20.9	0.02
	Male patients without previously known CAKUT		
	OR	95%CI	*P*
AKI	4.2	1.5–11.9	0.007
Megaureter	2.0	0.74–5.5	0.17
Non-*E. coli* infection	2.9	1.3–6.7	0.01
	Female patients with previously known CAKUT		
	OR	95%CI	*p*
AKI	20.1	1.4–276.1	0.02
	Female patients without previously known CAKUT		
	OR	95%CI	*p*
AKI	3.0	1.2–7.4	0.01

*AKI* Acute kidney injury, *CAKUT* Congenital anomalies of the kidney and urinary tract, *CI* Confidence interval, *fUTIs* Recurrent febrile urinary tract infections, *OR* Odds ratio

## Discussion

In this large nationwide cohort, we deliberately adopted the perspective of hospital pediatricians to identify clinically relevant factors associated with recurrent fUTIs that are readily identifiable during hospitalization in children aged 2 months to 3 years with fUTI, with the aim of providing information to support discharge planning. Older age, previously known CAKUT, AKI, and hydronephrosis with an APD of 5–15 mm were independently associated with recurrent fUTIs in the overall population.

Because CAKUT are among the most important factors associated with recurrent fUTIs, we performed additional analyses according to previously known CAKUT status and sex [1].

In these stratified analyses, phimosis was independently associated with recurrent fUTIs among males with previously known CAKUT, whereas AKI and non-*E. coli* infection were independently associated with recurrent fUTIs among males without previously known CAKUT. Among females, AKI was independently associated with recurrent fUTIs regardless of CAKUT status.

Children with recurrent infections were also more likely to be receiving antibiotic prophylaxis at admission, likely reflecting the presence of previously recognized risk factors for recurrence.

Overall, AKI emerged as the most consistent hospitalization-related factor, remaining independently associated with recurrent fUTIs in the overall cohort and across several clinically defined subgroups. Children with CAKUT may have a congenitally reduced nephron mass [13] and, consequently, reduced renal functional reserve, potentially increasing their susceptibility to AKI during stressful conditions such as fUTI [2]. Our group previously demonstrated that AKI occurring during fUTI may predict the presence of VUR [8]. Therefore, even mild AKI should not be overlooked during hospitalization for fUTI, as it may represent an indirect marker of reduced renal functional reserve and raise suspicion of an underlying CAKUT and help identify children with clinical features associated with recurrent fUTIs [2].

Similarly, non-*E. coli* infection may act as a clinical marker of underlying CAKUT and a higher baseline risk of recurrence [6, 9].

The association between phimosis and recurrence in males with previously diagnosed CAKUT further highlights the role of this local and potentially modifiable “urological factor” [14–17]. A systematic genital examination is simple, cost-free, and often underemphasized during inpatient evaluation. This assessment is clinically important because treatment of phimosis, either through circumcision or topical corticosteroids, has been associated with a reduced risk of fUTI recurrence [17].

Moreover, we found that all children receiving antibiotic prophylaxis before hospitalization belonged to the recurrent-fUTI group, whereas none of the children without recurrent fUTIs were receiving prophylaxis. This distribution resulted in complete separation and prevented the reliable estimation of its independent association in standard logistic regression. This finding should not be interpreted as evidence that prophylaxis increases the likelihood of recurrence, but most likely reflects confounding by indication, because prophylaxis had presumably been prescribed to children already considered at increased risk or with previous infections. Therefore, breakthrough fUTI during prophylaxis should raise suspicion of complex or previously undiagnosed CAKUT.

Constipation is another recognized factor associated with fUTI recurrence [18]. However, this information was not systematically collected in our cohort, and we could therefore not evaluate its contribution.

**Fig. 2:**
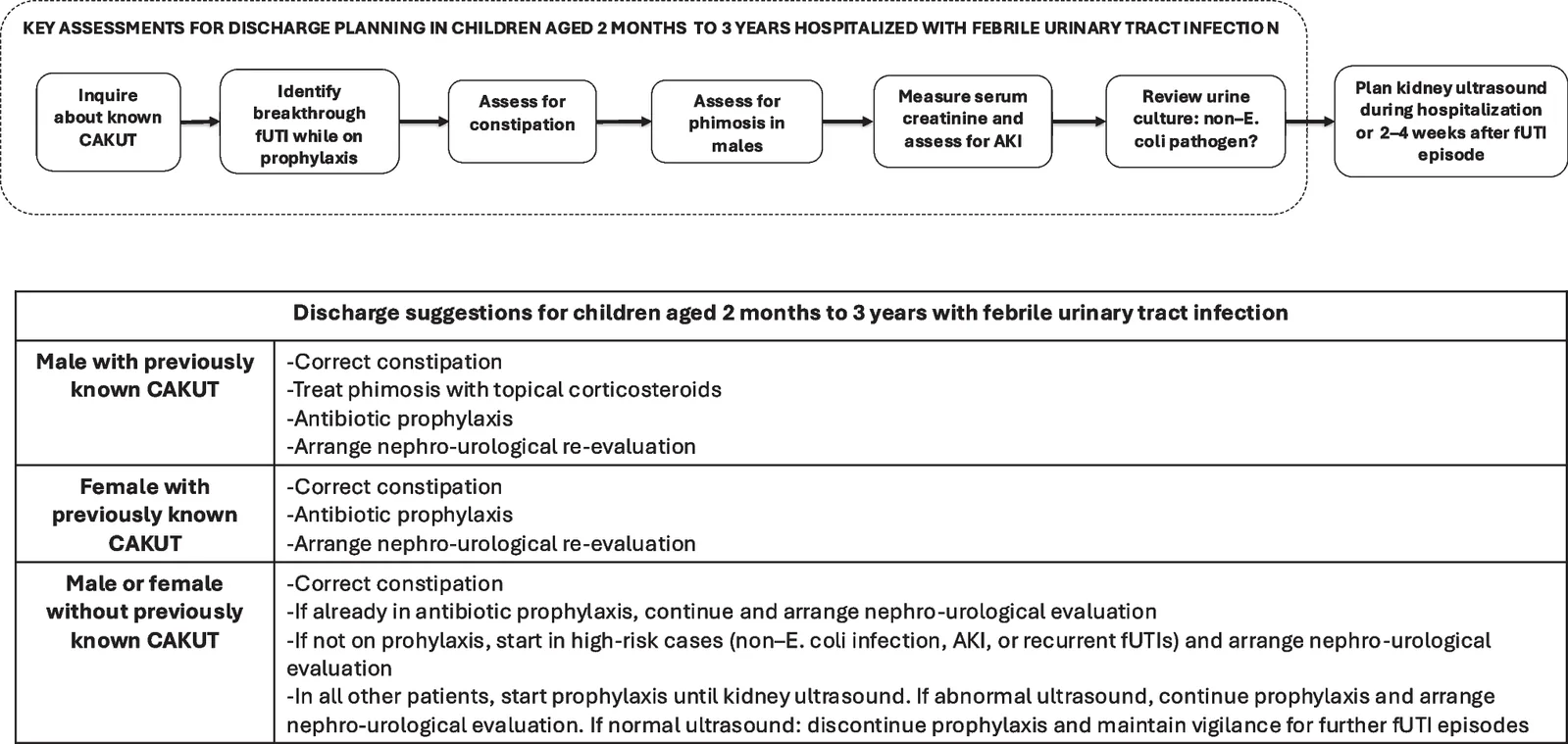
A framework for tailored discharge planning after pediatric febrile urinary tract infection. Abbreviations: AKI, acute kidney injury; CAKUT, congenital anomalies of the kidney and urinary tract; fUTI, febrile urinary tract infection

Current Italian recommendations provide guidance on the diagnosis and acute treatment of fUTI, the timing of kidney ultrasound, indications for further imaging, and nephro-urological referral, but offer limited guidance on how findings obtained during hospitalization should be integrated into individualized discharge planning [6, 9]. Building on our findings and the current body of evidence, we developed the discharge-oriented framework shown in Fig. 2 to illustrate how these elements may be integrated into tailored discharge planning after pediatric fUTI. The framework intentionally treats previously known CAKUT as a single category because reliable risk estimates for individual CAKUT phenotypes were not possible in the present cohort. Moreover, it is intended as a pragmatic tool to complement current recommendations and support individualized clinical assessment rather than as a prospectively validated management algorithm.

This study has several limitations. First, its retrospective design limits causal interpretation. Second, urine specimens were collected using different techniques, including sterile urine collection bags, which could have influenced diagnostic accuracy in a subset of patients. Third, baseline serum creatinine was not available for all children and was therefore retrospectively estimated in part of the cohort, although validated methods were used [8]. Fourth, information on some recognized risk factors for recurrent fUTIs, such as constipation, was not systematically collected and could not be included in the analyses. Finally, among patients with previously known CAKUT, it would have been informative to compare the risk of recurrent fUTI across individual CAKUT categories. However, the small number of patients in each category precluded reliable comparisons.

In conclusion, among children aged 2 months to 3 years admitted for fUTI, readily available clinical, laboratory, microbiological and imaging findings were associated with recurrent fUTIs. Our study complements current Italian recommendations [6, 9] by identifying hospitalization-related factors associated with recurrent fUTIs and integrating these findings with established evidence into a practical discharge-oriented framework. The proposed framework (Fig. 2) emphasizes the assessment of previously known CAKUT, AKI, non-*E. coli* infection, phimosis, constipation, and breakthrough fUTI during antibiotic prophylaxis and links these factors to practical considerations regarding modifiable risk factors, interim antibiotic prophylaxis, kidney ultrasound, and nephro-urological reassessment. Considering these elements during hospitalization may help hospital pediatricians identify children who may benefit from targeted interventions and tailored discharge planning.

## Supplementary Information

Below is the link to the electronic supplementary material.

Supplementary file 1 (DOCX 24.3 KB)

Supplementary file 2 (DOCX 22.6 KB)

## Data Availability

The datasets used and/or analysed during the current study are available from the corresponding author on reasonable request.

## Abbreviations

AKI: Acute kidney injury
CAKUT: Congenital anomalies of the kidney and urinary tract
fUTIs: Febrile urinary tract infections

## Code availability

N/A.
