# Supplementary material for: Tailoring discharge planning after pediatric febrile urinary tract infection: a cohort study
Source: Eur J Pediatr. 2026 Oct 13;185(11):841. doi: 10.1007/s00431-026-07479-0 (PMC13661712; doi:10.1007/s00431-026-07479-0)
Supplement: Supplementary file 1 [file 431_2026_7479_MOESM1_ESM.docx]

**Supplementary Table 1. General characteristics of male patients with and without recurrent fUTIs on the basis of the presence or not of previously known CAKUT.**

| **Presence of previously known CAKUT** | | | | |
| --- | --- | --- | --- | --- |
|  | **Recurrent fUTIs (Yes) No.= 30** | **Recurrent fUTIs (No) No.= 23** | **p** |  |
| **Age, years, mean (SDS)** | 1.2 (0.88) | 0.9 (0.85) | 0.26 |  |
| **Birth weight, kg, median (IQR)** | 3.3 (0.50) | 3.1 (0.78) | 0.49 |  |
| **Preterm birth, No. (%)** | 2 (6.7) | 5 (21.7) | 0.22 |  |
| **CKD, No. (%)** | 2 (6.7) | 0 (0) | 0.49 |  |
| **Antibiotic prophylaxis No. (%)** | 22 (73.3) | 0 (0) | **<0.001** |  |
| **Phimosis, No. (%)** | 13 (43.3) | 3 (13.0) | **0.03** |  |
| **Highest/basal serum creatinine ratio, median (IQR)** | 1.3 (0.8) | 1.0 (1.0) | 0.32 |  |
| **AKI, No. (%)** | 9 (30.0) | 6 (26.1) | 0.75 |  |
| **Na, mEq/L, mean (SDS)** | 135.9 (3.5) | 136.9 (3.0) | 0.27 |  |
| **Hyponatremia, No. (%)** | 6 (20.0) | 4 (17.4) | 0.99 |  |
| **Hypernatremia, No.(%)** | 0 (0) | 0 (0) | N/A |  |
| **K, mEq/L, mean (SDS)** | 4.5 (0.47) | 4.9 (0.74) | **0.02** |  |
| **Hyperkalemia, No. (%)** | 0 (0) | 2 (8.7) | 0.18 |  |
| **Hypokalemia, No. (%)** | 0 (0) | 0 (0) | N/A |  |
| **Hyponatremia and hyperkalemia, No. (%)** | 2 (6.7) | 1 (4.3) | 0.99 |  |
| **WBC, n/mcL, median (IQR)** | 14960 (5730) | 15170 (9637) | 0.32 |  |
| **Neutrophils n/mcL, median (IQR)** | 8310 (5134) | 8595 (7205) | 0.65 |  |
| **CRP, mg/dL, mean (SDS)** | 12.7 (15.4) | 12.6 (24.0) | 0.98 |  |
| **Platelets, n/mcL, median (IQR)** | 375000 (218000) | 365000 (245000) | 0.50 |  |
| **Hydronephrosis (APD 5-15 mm), No. (%)** | 10 (33.3) | 2 (8.7) | 0.048 |  |
| **Hydronephrosis (APD >15 mm), No. (%)** | 10 (33.3) | 9 (39.1) | 0.77 |  |
| **Megaureter, No. (%)** | 17 (56.7) | 14 (60.9) | 0.78 |  |
| **Kidney Hypodysplasia, No. (%)** | 7 (23.3) | 9 (39.1) | 0.21 |  |
| **Bladder abnormalities, No. (%)** | 0 (0) | 1 (4.3) | 0.43 |  |
| **Non-E. coli infection, No. (%)** | 13 (43.3) | 13 (56.5) | 0.34 |  |
| **Absence of previously known CAKUT** | | | | |
|  | **Recurrent fUTIs (Yes) No.=38** | **Recurrent fUTIs (No) No.=214** | **p** |  |
| **Age, years, mean (SDS)** | 1.1 (1.0) | 0.63 (0.68) | **<0.001** |  |
| **Birth weight, kg, median (IQR)** | 3.25 (0.76) | 3.3 (0.73) | 0.98 |  |
| **Preterm birth, No. (%)** | 9 (23.7) | 34 (15.9) | 0.25 |  |
| **CKD, No. (%)** | 1 (2.6) | 4 (1.9) | 0.56 |  |
| **Antibiotic prophylaxis No. (%)** | 10 (26.3) | 0 (0) | **<0.001** |  |
| **Phimosis, No. (%)** | 10 (26.3) | 39 (18.2) | 0.27 |  |
| **Highest/basal serum creatinine ratio, median (IQR)** | 1.0 (0.66) | 0.81 (0.36) |  |  |
| **AKI, No. (%)** | 10 (26.3) | 10 (4.7) | **<0.001** |  |
| **Na, mEq/L, mean (SDS)** | 137.0 (3.4) | 136.1 (2.8) | 0.10 |  |
| **Hyponatremia, No. (%)** | 9 (23.7) | 49 (22.9) | 0.91 |  |
| **Hypernatremia, No.(%)** | 0 (0) | 0 (0) | N/A |  |
| **K, mEq/L, mean (SDS)** | 4.65 (0.75) | 4.86 (0.61) | 0.07 |  |
| **Hyperkalemia, No. (%)** | 3 (7.9) | 15 (7.0) | 0.85 |  |
| **Hypokalemia, No. (%)** | 0 (0) | 1 (0.5) | 0.99 |  |
| **Hyponatremia and hyperkalemia, No. (%)** | 1 (2.6) | 6 (2.8) | 0.99 |  |
| **WBC, n/mcL, median (IQR)** | 16400 (9905) | 16010 (9030) | 0.99 |  |
| **Neutrophils n/mcL, median (IQR)** | 9470 (6480) | 8380 (6650) | 0.18 |  |
| **CRP, mg/dL, mean (SDS)** | 30.3 (77.6) | 19.2 (34.9) | 0.16 |  |
| **Platelets, n/mcL, median (IQR)** | 328500 (212500) | 416000 (185000) | 0.06 |  |
| **Hydronephrosis (APD 5-15 mm), No. (%)** | 6 (15.8) | 27 (12.6) | 0.60 |  |
| **Hydronephrosis (APD >15 mm), No. (%)** | 6 (15.8) | 15 (7.0) | 0.07 |  |
| **Megaureter, No. (%)** | 8 (21.1) | 18 (8.4) | **0.02** |  |
| **Kidney Hypodysplasia, No. (%)** | 4 (10.5) | 12 (5.6) | 0.27 |  |
| **Bladder abnormalities, No. (%)** | 0 (0) | 0 (0) | N/A |  |
| **Non-E. coli infection, No. (%)** | 14 (36.8) | 27 (12.6) | **<0.001** |  |

*Abbreviations*: AKI, acute kidney injury; APD, anterior-posterior diameter; CAKUT, congenital anomalies of the kidney and urinary tract; CKD, chronic kidney disease; CRP, c-reactive protein; fUTIs, febrile urinary tract infections; IQR, interquartile range; Na, sodium; K, potassium; SDS, standard deviation score; WBC, white blood cells.
