# Supplementary material for: Tailoring discharge planning after pediatric febrile urinary tract infection: a cohort study
Source: Eur J Pediatr. 2026 Oct 13;185(11):841. doi: 10.1007/s00431-026-07479-0 (PMC13661712; doi:10.1007/s00431-026-07479-0)
Supplement: Supplementary file 2 [file 431_2026_7479_MOESM2_ESM.docx]

**Supplementary Table 2. General characteristics of female patients with and without recurrent fUTIs on the basis of the presence or not of previously known CAKUT.**

| **Presence of previously known CAKUT** | | | |
| --- | --- | --- | --- |
|  | **Recurrent fUTIs (Yes) No.= 17** | **Recurrent fUTIs (No) No.= 12** | **p** |
| **Age, years, mean (SDS)** | 1.15 (0.92) | 1.1 (1.3) | 0.89 |
| **Birth weight, kg, median (IQR)** | 3.1 (0.85) | 3.2 (0.93) | 0.68 |
| **Preterm birth, No. (%)** | 5 (29.4) | 1 (8.3) | 0.35 |
| **CKD, No. (%)** | 1 (5.9) | 1 (8.3) | 0.99 |
| **Antibiotic prophylaxis No. (%)** | 5 (29.4) | 0 (0) | 0.06 |
| **Highest/basal serum creatinine ratio, median (IQR)** | 1.6 (0.78) | 0.97 (0.66) | 0.26 |
| **AKI, No. (%)** | 9 (52.9) | 1 (8.3) | **0.02** |
| **Na, mEq/L, mean (SDS)** | 135.6 (3.2) | 135.0 (3.9) | 0.64 |
| **Hyponatremia, No. (%)** | 7 (41.2) | 2 (16.7) | 0.23 |
| **Hypernatremia, No.(%)** | 0 (0) | 0 (0) | N/A |
| **K, mEq/L, mean (SDS)** | 4.9 (0.68) | 5.2 (0.80) | 0.18 |
| **Hyperkalemia, No. (%)** | 2 (11.8) | 2 (16.7) | 0.99 |
| **Hypokalemia, No. (%)** | 0 (0) | 0 (0) | N/A |
| **Hyponatremia and hyperkalemia, No. (%)** | 0 (0) | 3 (25.0) | 0.06 |
| **WBC, n/mcL, median (IQR)** | 12780 (14930) | 17390 (9420) | 0.99 |
| **Neutrophils n/mcL, median (IQR)** | 5500 (12390) | 9950 (4720) | 0.43 |
| **CRP, mg/dL, mean (SDS)** | 24.9 (60.8) | 37.0 (78.8) | 0.65 |
| **Platelets, n/mcL, median (IQR)** | 333000 (154000) | 468000 (449000) | 0.99 |
| **Hydronephrosis (APD 5-15 mm), No. (%)** | 3 (17.6) | 4 (33.3) | 0.40 |
| **Hydronephrosis (APD >15 mm), No. (%)** | 1 (5.9) | 1 (8.3) | 0.80 |
| **Megaureter, No. (%)** | 4 (23.5) | 6 (50.0) | 0.24 |
| **Renal Hypodysplasia, No. (%)** | 3 (17.6) | 3 (25.0) | 0.67 |
| **Bladder abnormalities, No. (%)** | 1 (5.9) | 1 (8.3) | 0.99 |
| **Non-E. coli infection, No. (%)** | 10 (58.8) | 6 (50.0) | 0.72 |
| **Absence of previously known CAKUT** | | | |
|  | **Recurrent fUTIs (Yes) No.= 42** | **Recurrent fUTIs (No) No.= 194** | **p** |
| **Age, years, mean (SDS)** | 1.5 (1.1) | 1.0 (0.89) | 0.001 |
| **Birth weight, kg, median (IQR)** | 3.1 (0.68) | 3.2 (0.53) | 0.76 |
| **Preterm birth, No. (%)** | 6 (14.3) | 29 (14.9) | 0.91 |
| **CKD, No. (%)** | 2 (4.8) | 1 (0.5) | 0.08 |
| **Antibiotic prophylaxis No. (%)** | 7 (16.7) | 0 (0) | **<0.001** |
| **Highest/basal serum creatinine ratio, median (IQR)** | 1.1 (0.49) | 0.92 (0.45) | **0.01** |
| **AKI, No. (%)** | 9 (21.4) | 16 (8.2) | **0.01** |
| **Na, mEq/L, mean (SDS)** | 136.3 (2.6) | 136.5 (3.2) | 0.69 |
| **Hyponatremia, No. (%)** | 12 (28.6) | 46 (23.7) | 0.51 |
| **Hypernatremia, No.(%)** | 0 (0) | 2 (1.0) | 0.99 |
| **K, mEq/L, mean (SDS)** | 4.5 (0.57) | 4.7 (0.57) | 0.12 |
| **Hyperkalemia, No. (%)** | 0 (0) | 9 (4.6) | 0.37 |
| **Hypokalemia, No. (%)** | 0 (0) | 2 (1.0) | 0.99 |
| **Hyponatremia and hyperkalemia, No. (%)** | 1 (2.4) | 1 (0.5) | 0.32 |
| **WBC, n/mcL, median (IQR)** | 17850 (14240) | 16535 (7465) | 0.86 |
| **Neutrophils n/mcL, median (IQR)** | 9570 (14110) | 8635 (6655) | 0.99 |
| **CRP, mg/dL, mean (SDS)** | 8.7 (12.9) | 7.8 (10.2) | 0.12 |
| **Platelets, n/mcL, median (IQR)** | 455500 (186250) | 396000 (191500) | 0.66 |
| **Hydronephrosis (APD 5-15 mm), No. (%)** | 4 (9.5) | 10 (5.2) | 0.28 |
| **Hydronephrosis (APD >15 mm), No. (%)** | 3 (7.1) | 10 (5.2) | 0.71 |
| **Megaureter, No. (%)** | 2 (4.8) | 4 (2.1) | 0.29 |
| **Renal Hypodysplasia, No. (%)** | 4 (9.5) | 5 (2.6) | 0.06 |
| **Bladder abnormalities, No. (%)** | 1 (2.4) | 1 (0.5) | 0.32 |
| **Non-E. coli infection, No. (%)** | 6 (14.3) | 24 (12.4) | 0.80 |

*Abbreviations*: AKI, acute kidney injury; APD, anterior-posterior diameter; CAKUT, congenital anomalies of the kidney and urinary tract; CKD, chronic kidney disease; CRP, c-reactive protein; fUTIs, febrile urinary tract infections; IQR, interquartile range; Na, sodium; K, potassium; SDS, standard deviation score; WBC, white blood cells.
